# Adaptive Flexibility of Oldowan Hominins: Secondary Use of Flakes at Bizat Ruhama, Israel

**DOI:** 10.1371/journal.pone.0066851

**Published:** 2013-06-20

**Authors:** Yossi Zaidner

**Affiliations:** 1 Zinman Institute of Archaeology, University of Haifa, Haifa, Mount Carmel, Israel; 2 Institute of Archaeology, The Hebrew University of Jerusalem, Mt. Scopus, Jerusalem, Israel; University of Oxford, United Kingdom

## Abstract

The lithic assemblage of the Early Pleistocene site of Bizat Ruhama, Israel demonstrates the earliest evidence for systematic secondary knapping of flakes. The site, dated to the Matuyama chron, is one of the earliest primary context Oldowan occurrences in Eurasia. According to the experimental replication of the stone-tool production sequence, the secondary knapping of flakes was a part of a multi-stage operational sequence targeted at the production of small (<2 cm) flakes. This sequence included four stages: acquisition of chert pebbles, production of flakes, deliberate selection of flakes of specific morphologies, and their secondary knapping by free-hand or bipolar methods. The results suggest that flakes with retouch-like scars that were produced during this sequence and which commonly are interpreted as shaped tools are unintentional waste products of the small flake production. The intentional manufacture of very small flakes at Bizat Ruhama was probably an economic response to the raw material constrains. Systematic secondary knapping of flakes has not yet been reported from other Early Pleistocene sites. Systematic secondary knapping for small flake production became increasingly important only in the lithic industries of the second half of the Middle Pleistocene, almost a million years later. The results from Bizat Ruhama indicate that Oldowan stone-tool production sequence was conceptually more complex than previously suggested and offer a new perspective on the capabilities for invention and the adaptive flexibility of the Oldowan hominins.

## Introduction

The lithic industries assigned to the Oldowan techno-complex are characterized by production of unmodified sharp-edged flakes [1], [2], [3], [4]. These sharp flakes were obtained by various methods, some of which indicate that as early as 2.34 Myr ago hominins had developed knapping skills and manual dexterities that allowed them to organize and predetermine the knapping and produce long sequences of flaking [1]. Although technically well developed, conceptually these industries represent simple two-stage operational schemes consisting of raw material acquisition followed by detachment of the flakes (e.g. [1], [2], [4], [5], [6], [7], [8], [9], [10], [11], [12], [13], [14]). Longer, more complex sequences have been associated with the Acheulian techno-complex (e.g. [3], [15], [16], [17]).

This paper reports on systematic secondary flake knapping recorded at the Early Pleistocene site of Bizat Ruhama, Israel, indicating that Oldowan hominins employed more complex operational schemes than previously suggested. The site of Bizat Ruhama, currently dated to 1.6-1.2 Ma on the basis of biochronological and paleomagnetic considerations [18], [19], is one of the earliest primary context hominin sites in Eurasia. The excavations yielded several lithic assemblages (ca. 2700 artifacts), predominantly cores and debitage products. No traces of Acheulian biface production or any other form of bifacial or discoidal knapping was found. The characteristics of the core technology and the chronological context suggest that Bizat Ruhama belongs to the Oldowan techno-complex [19], [20].

Approximately half of the flakes that were produced during core reduction were subsequently further flaked, broken or notched, most commonly resulting in Clactonian notches or flakes with retouch-like abrupt scars. These types of thick modified flakes are the most distinct product of the Bizat Ruhama industry, distinguishing it from other Early Pleistocene assemblages. Originally the modified flakes in Bizat Ruhama were interpreted as retouched tools [21], [22], however later revision of the material in conjunction with study of the new assemblages excavated in 2004–05 cast doubt on this classification for several reasons. Firstly, signs of notching, breakage and flaking often occur together on the same flake. Secondly, many of these flakes exhibit percussion marks at the point of intersection between the dorsal and lateral surfaces opposite to the retouch-like scars/Clactonian notch. It has been suggested previously that similar marks at the impact point of the hammerstone are characteristic of flakes breaking on an anvil, and that occasionally the anvil impact will also cause accidental ‘spontaneous retouch’ [23], [24]. Such spontaneous removals often have the shape of a Clactonian notch or of a series of Clactonian notches, and can be confused with intentional retouch [23], [25].

An experimental study was therefore undertaken in order to test the hypothesis that an anvil have been used in the modification of flakes at Bizat Ruhama. Flakes were detached from local pebbles, and then further knapped on an anvil. The results of the experimental knapping suggest that the majority of the modified flakes at Bizat Ruhama are waste products of the process of manufacturing small, thin and sharp flakes on an anvil. The results indicate that the production sequence at Bizat Ruhama consisted of more stages and was conceptually more complex than previously reported for Oldowan sites. The study points to a high level of technological and adaptive flexibility of the Early Pleistocene hominins on the threshold of Eurasia.

## Materials and Methods

### The Site and the Assemblages

**Figure 1:**
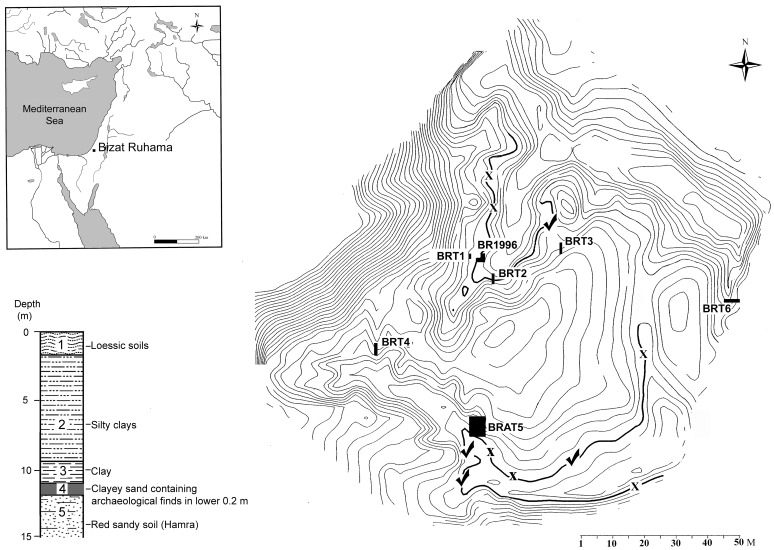
Map, plan and stratigraphic column of Bizat Ruhama site. BR1996– Bizat Ruhama, area excavated in 1996. BRAT5, BRT1, BRT2, BRT3, BRT4, BRT6–2004–05 excavated areas and trenches. γ– Sampled locations with *in situ* artifacts or bones. X – Sampled locations without artifacts or bones. -Thick curved lines mark the contour of the erosional channels along which archaeological layer is exposed. Stratigraphy: 1. 01.–2 m. Loessial arid brown soil; 2. 11–12 m. Brown silty clays/grumosol; the basal 3 m are situated within the Matuyama reverse polarity chron (1.96–0.78 Ma). 3. 1–3 m. Grayish black, massive, prismatic, greasy clay/loam; extensive iron–manganese impregnation; dated to the Matuyama reverse polarity chron (1.96–0.78 Ma). Palustrine origin (seasonal ponds). 4. 0.2–1 m. Massive sand with some clayey domains; archaeological remains in lower 0.2 m; dated to the Matuyama reverse polarity chron (1.96–0.78 Ma); locally reworked sand and soil aggregates from the Stratum 5 topsoil by wind and overland flow. 5. Unknown depth. Red sandy soil (locally known as *hamra*) formed on coastal sand dunes; archaeological bones and lithics within the uppermost 5 cm and at the interface with Stratum 4; dated to the Matuyama reverse polarity chron (1.96–0.78 Ma).

Bizat Ruhama is a single-horizon open-air site on the fringe of the Negev Coastal Plain, 25 km east of the present-day Mediterranean coastline of Israel (Figure 1). The archaeological horizon was discovered at the bottom of two erosional channels on the edge of one of the badland fields that are typical of this part of the Coastal Plain (all necessary permits were obtained for the described field studies. The permits were obtained from Department of Excavations and Surveys, Israel Antiquities Authority, P.O. Box 586, Jerusalem 91004; permission numbers G-49 (2004), G-42 (2005)). Erosion exposed a depositional sequence ca. 17 m thick, composed of loessic sediments, clays and sands [19], [26]. The archaeological material was found at the bottom of the sandy layer (Stratum 4; Figure 1) overlying a red sandy loam (locally known as hamra; Stratum 5). The sandy layer is covered by dark black clay (Stratum 3). In all the excavated areas artifacts and bones appear in the lower part of the sandy layer close to, or immediately on the contact with the underlying hamra [19], [21], [26]. The finds occur in patches of variable densities over an area of a few thousand square meters. According to geological and micromorphological studies, hominins occupied the surface of the *hamra* in an undulating inter-dune depression. The clayey sand of the archaeological layer represents input of locally reworked sand and soil aggregates from the *hamra* topsoil by low energy deposition, possibly through wind and overland flow. According to micromorphological studies the archaeological assemblages are in primary context and represent either a single occupation or several occupation events within a relatively short period of time [26].

The faunal assemblage at Bizat Ruhama includes mainly equids (*Equus cf. tabeti*), followed by antelopes (*Pontoceros ambiguous/Spirocerus* sp.), bovids (cf. *Bison* sp.) and gazelles (*Gazella gazella*), all representative of an open environment with patchy water sources [27]. Both sedimentological and faunal evidence indicate hominin exploitation of an open, poorly vegetated, semi-arid environment.

The archaeological horizon at Bizat Ruhama was dated to the Matuyama reversed polarity chron (1.96-0.78 mya; [21], [28]). The presence of *Equus cf. tabeti*, and antelope *Pontoceros ambiguus* or *Spirocerus* sp. in the faunal assemblage also points to the Early Pleistocene age of the site [27]. Recently it was suggested that the fauna of Bizat Ruhama belongs to the same faunal unit as Ubeidiya and predates the Jaramillo normal event. The site is thus likely to be roughly contemporaneous to Ubeidya, which is currently dated to ca 1.6-1.2 Ma [18].

The lithic finds from Bizat Ruhama comprise five assemblages (n = 1958 artifacts). The majority of the artifacts (N = 1694) derive from excavation areas BRAT5 and BR1996, and the rest were unearthed in three excavated trenches: BRT1, BRT2 and BRT3 (Figure 1). A large assemblage of ca. 700 artifacts was collected from surface exposures of the archaeological layer.

The raw material used at the site consists of small, rounded chert pebbles from the nearby Pleshet Formation [20], [29]. Both the bipolar and the freehand hard hammer technique were used during the knapping of the pebbles. The choice of methods and techniques was largely determined by the size and shape of the available pebbles: small pebbles were knapped by the bipolar technique [19], [20], while larger pebbles were often knapped by more organized methods, including the unidirectional orthogonal (or abrupt [30]) method in which a series of 2–5 flakes was removed from a single platform. The unidirectional orthogonal method was mostly used on single-platform cores, although in some cases the cores were rotated and an additional series of flakes was removed from other platforms, resulting in cores of polyhedral and subspheroid shape [19]. Although some large cobbles were used at the site (up to 28 cm; 3.8 kg), flakes larger than 6 cm were not produced and the average length of the flakes is 25 mm. In addition, neither bifacial nor discoidal knapping methods were used at the site, and traces of biface production technology, which is abundant in Acheulian assemblages in the site’s area [27], [31], [32], were not found.

### Methodology for the Lithic Studies

The Bizat Ruhama lithic industry was studied using experimental and technological approaches. The comprehensive experimental knapping program at the site included free-hand hard hammer and bipolar flaking of pebbles, an anvil breakage of flat pebbles and free-hand and anvil knapping/breakage of flakes [31]. This paper presents the results of the experiments in knapping/breakage of the flakes on an anvil (henceforth bipolar reduction technique) aimed at reconstruction of the methods and goals of the secondary flake knapping.

For the controlled knapping experiments, 65 flakes were selected among the flakes produced during the experimental pebble reduction. The selection criteria were based on the features of secondary knapped flakes in the archaeological assemblages. Thus, large and thick flakes, whose lateral edges had an angle of 50–80 degrees, were selected. The bipolar reduction procedure followed the general rules recorded in ethnographic and experimental studies. The bipolar technique consists of placing the core/flake on an anvil and striking it along a roughly perpendicular plane [33], [34], [35], [36], [37], [38], [39]. In the course of the experiments, the flakes were placed with their flat (usually ventral) surface on the anvil and held between the thumb and the fingers. The blow was delivered near the mesial part of the flake, far from the edges. When possible, the flakes were hand-supported. Smaller flakes were simply smashed with a hammerstone, with no hand-support. The list of observations taken before, during and after the knapping is presented in the supporting information (Table S1). One hammerstone (rounded chert cobble weighing 415 gr, 8 cm long) and one anvil (flat chert cobble 22 cm long, 16 cm wide and 9 cm thick) were used during the entire experiment.

The products of experimental knapping were compared to secondary knapped flakes in archaeological assemblages using the same list of technological, morphological and metrical attributes (Table S2).

## Results

### Archaeological Data

**Figure 2:**
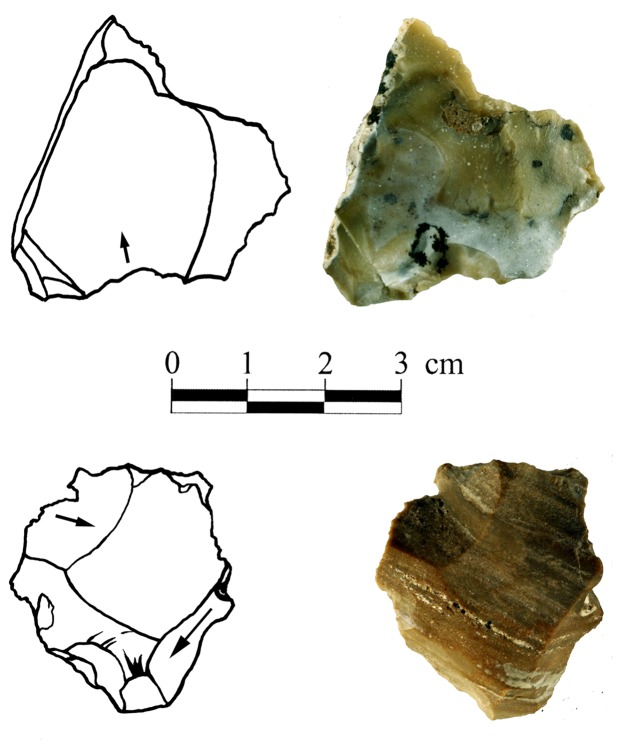
Cores-on-flake in Bizat Ruhama archaeological assemblages.

**Figure 3:**
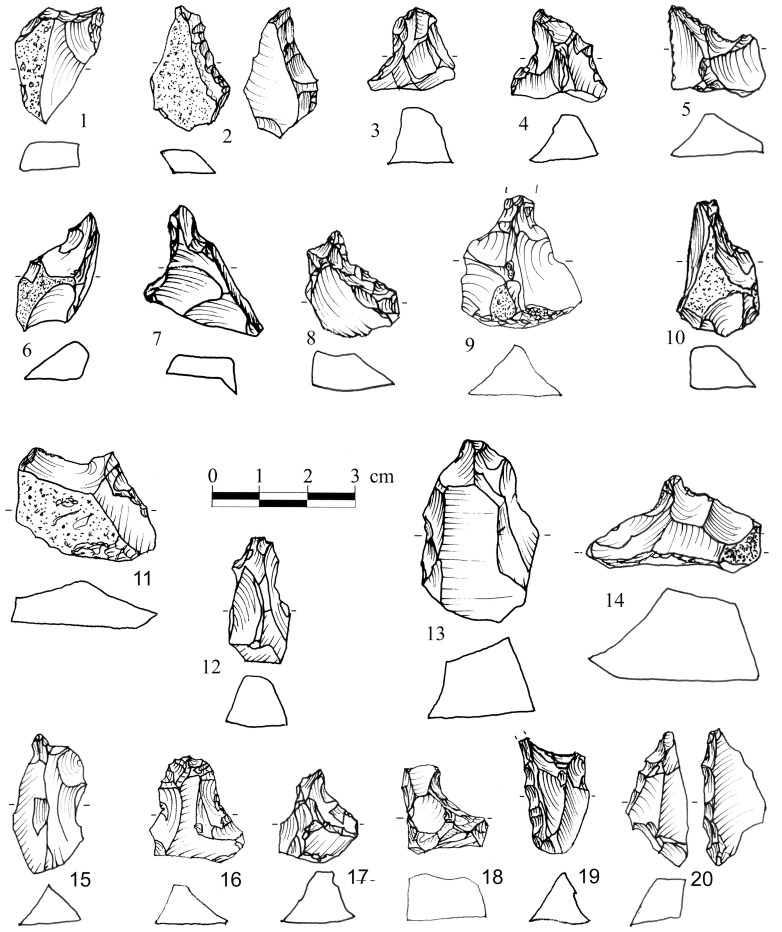
Secondary knapped flakes in Bizat Ruhama archaeological assemblages.

**Figure 4:**
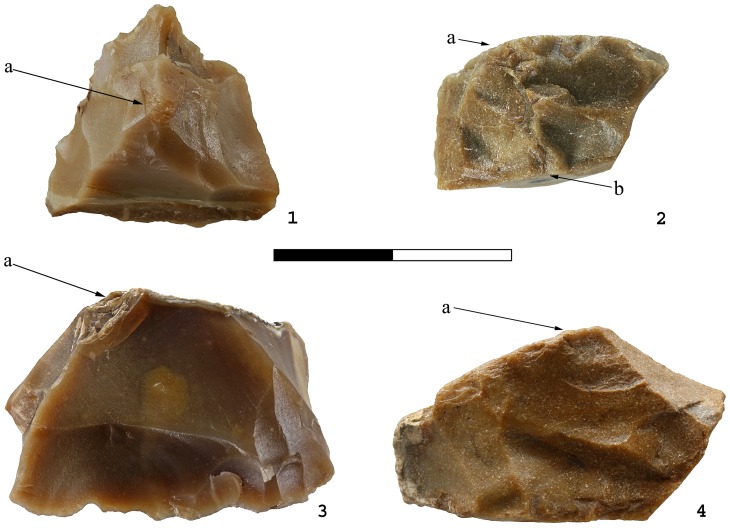
Secondary knapped flakes with the signs of dorsal impact in Bizat Ruhama assemblages. 1. Pointed piece with (a) point of percussion, crushing and crack-lines on the dorsal surface; 2. Broken flake with (a) signs of impact and crushing directed from the dorsal surface, (b) signs of impact and crushing directed from the ventral surface; 3. Clactonian notch with (a) opposite point of percussion and crushing on the dorsal surface; 4. Flake with retouch-like scars with (a) opposite scars and crushing on the dorsal surface directed from the ventral surface.

Apart from a small number of flakes that were used as simple cores, from which 1–4 small flakes were removed (Figure 2), the flake assemblage includes Clactonian notches, pointed or modified flakes with irregular retouch-like scars and broken flakes with signs of dorsal impact (Table 1; Figure 3). The retouch-like scars often exhibit step fracture terminations. Step fracture scars are always wider than they are long and usually do not exhibit a clear negative of the bulb of percussion. They are common when the angle between the ventral and the broken/lateral surfaces is 80 degrees or steeper. Broken flakes often exhibit isolated step scars or signs of crushing where the ventral surface intersects with the broken or with the lateral surface of the flake (Figure 4). The flakes were usually broken on their mesial part, close to the point of maximum thickness.

**Table 1:** Secondary knapped flakes in Bizat Ruhama archaeological assemblages.

Cores-on-flake	58
Clactonian notches	218
Flakes with retouch-like scars	239
Pointed pieces	133
Broken flakes with signs of dorsal impact	177

The secondary knapped flakes often show impact marks at the intersection between the dorsal surface and the lateral or broken surfaces. The most frequent impact marks on the dorsal surfaces are points of percussion, crushing of the dorsal edges and crack lines (Figure 4).

**Figure 5:**
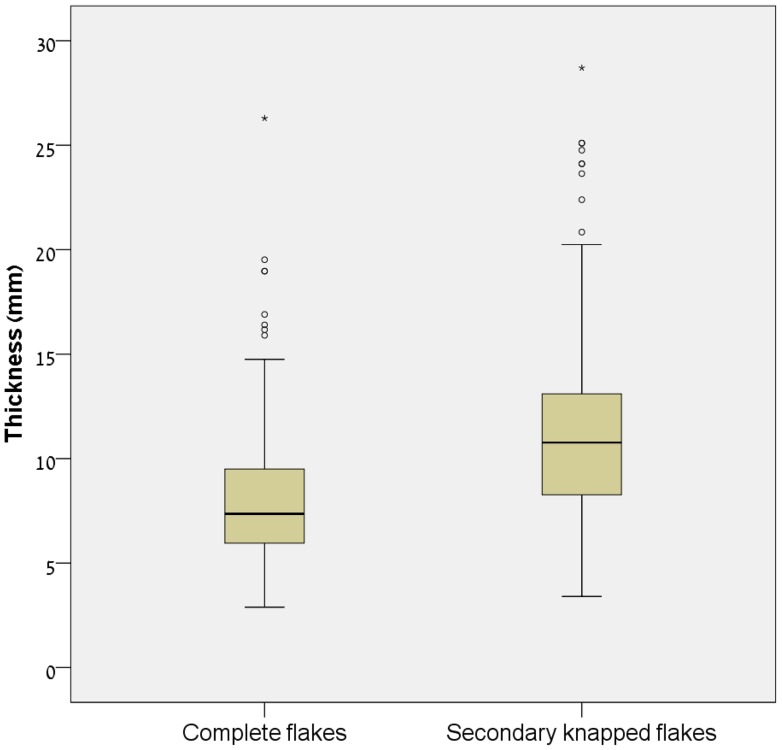
Thickness of complete flakes and secondary knapped flakes in Bizat Ruhama archaeological assemblages.

Flakes selected for secondary knapping are considerably thicker than complete flakes. Flakes thinner than 7 mm constitute approximately 45% of the complete flakes, but only 11.5% of secondary knapped flakes (Figure 5). These notable differences point at intentional selection of thick blanks and they are the reason that the edges of modified flakes are very steep.

Between 83 and 95% of the secondary knapped flakes from different excavation areas are fresh and unrolled [19], [26]. Abraded or slightly abraded artifacts were not included in the sample. The scars on the artifacts’ edges do not resemble natural retouch, since they often show clear signs of impact (crushing and incipient cones) and conchoidal fracture. Moreover, signs of impact on the dorsal faces are not expected to occur in naturally broken flakes. Broken flakes that show no signs of dorsal impact were not included in the sample, since we assume that the breakage may have resulted from knapping accidents, use, or post-depositional disturbances. Another argument in favor of the artificial origin of the scars is that they occur only on thick flakes, while if they resulted from the depositional disturbances, one would expect to find similar features on other artifact categories (cores, chunks, thin flakes, fragments, etc.).

### Experimental Data

#### The experiment

Bipolar technique flaking conceived as having fracture mechanics associated with the type of initiation called wedging [40]. Unlike Hertzian initiation, which is characterized by easily recognizable features such as bulb of percussion and ripples, the typical products of wedging initiation exhibit flat “shear” fracture surfaces. Wedging often does not leave marks on the ventral surface, sometimes up to the point that the ventral surface cannot be distinguished at all [40]. As a consequence, the industries in which bipolar technique was frequently used are dominated by broken fragments with shear fracture surfaces, without identifiable ventral surfaces, bulbs of percussion and butts [7], [33], [35], [36], [37], [38], [41].

Wedging initiation, however, will not necessary be generated while applying bipolar loading. According to Cotterell and Kamminga [40], the conditions necessary for the development of wedging initiation are an edge angle of 90 degrees or greater and placement of the blow well away from the edge of the core. Since these conditions are often fulfilled during bipolar reduction, in bipolar flakes the fracture typically starts with a wedging initiation. If, on the other hand, the angle of the edge is less than 90 degrees and the blow is delivered close to the core’s edge, there is a good chance that a Hertzian initiation will develop even in bipolar knapping. The variability recorded in bipolar-technique flake assemblages [33], [35], [36], [37], [38], [41], [42], [43] is probably connected to the fact that two types of initiation may occur during bipolar reduction.

Another defining feature of bipolar flaking is that the loading is applied to two opposite surfaces and may result in fractures in opposite directions – the flake can be detached by either a blow from a hammer or a counterblow from an anvil [36], [38], [40], [43], [44], [45]. The type of initiation and the direction from which the flake is detached vary according to the morphology of the blank and the way in which it was placed on an anvil. The features generally accepted as typical of bipolar knapping (shear fracture surfaces, absence of recognizable butts and bulbs, split flakes and angular fragments) generally occur when the angles between the debitage surface and the two opposite striking surfaces are close to 90 degrees (orthogonal surfaces) and the blow and counterblow occur on the same axis, leading to detachment of flakes from both surfaces (see discussion in Verges and Olle [43]).

**Figure 6:**
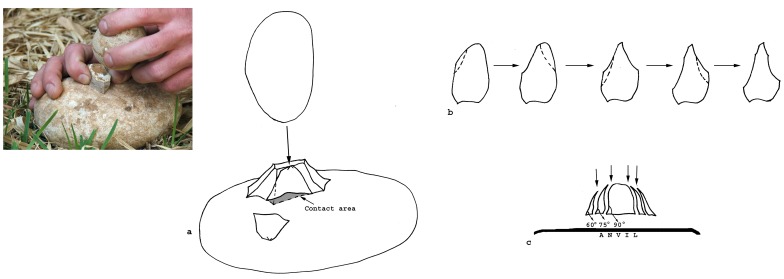
Schematic representation of the experiment in anvil-supported knapping of the flakes. a. The flake is rested with the ventral surface on the anvil. When the hammerstone hits the flake, small flakes are detached by an anvil impact from different edges of the flake. Because of the large contact area with the anvil, the removed flakes have large butts. Because of the relatively acute angle between the ventral and lateral surfaces the flakes have prominent bulbs of percussion caused by the anvil impact. At the contact between the hammerstone and the dorsal surface of the flake signs of impact are visible; b. View from above. c. View in the section. The flake that is actually converted into a core can change its shape during the knapping sequence from Clactonian notch to pointed piece etc., until the edges become too abrupt.

**Figure 7:**
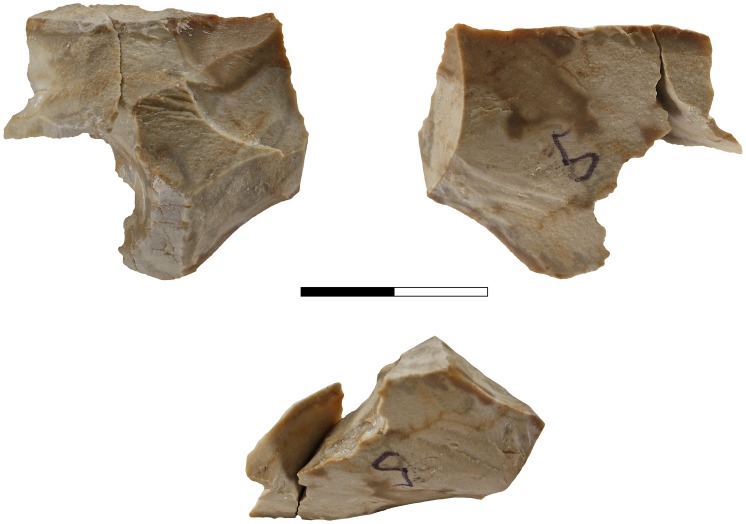
Experimental assemblages. **Clactonian notch conjoined with small flake that was detached during the knapping.** The flake was placed with the ventral surface on an anvil and struck on the dorsal surface with chert pebble hammerstone.

In the current experiment, the morphology of the knapped blank dictated different fracture mechanics. The angle between the ventral surface of the flake resting on an anvil and the flake edges was less than 90 degrees and the fracture was initiated mostly by anvil impact, usually close to the flake edge. The bipolar loading did not split or break the flake; rather, the flake functioned as a core and flakes and fragments were detached from its edges (Figure 6). The scars created by the anvil impact have all the characteristics of conchoidal fracture, namely negatives of the bulb of percussion, ripple marks, impact points and incipient cones of percussion, indicating that the fracture was initiated by a Hertzian initiation. It is likely that a combination of the thickness of the flakes and the acute angle at which their edges met the anvil surface facilitated the Hertzian initiation (Figures 6; 7).

**Figure 8:**
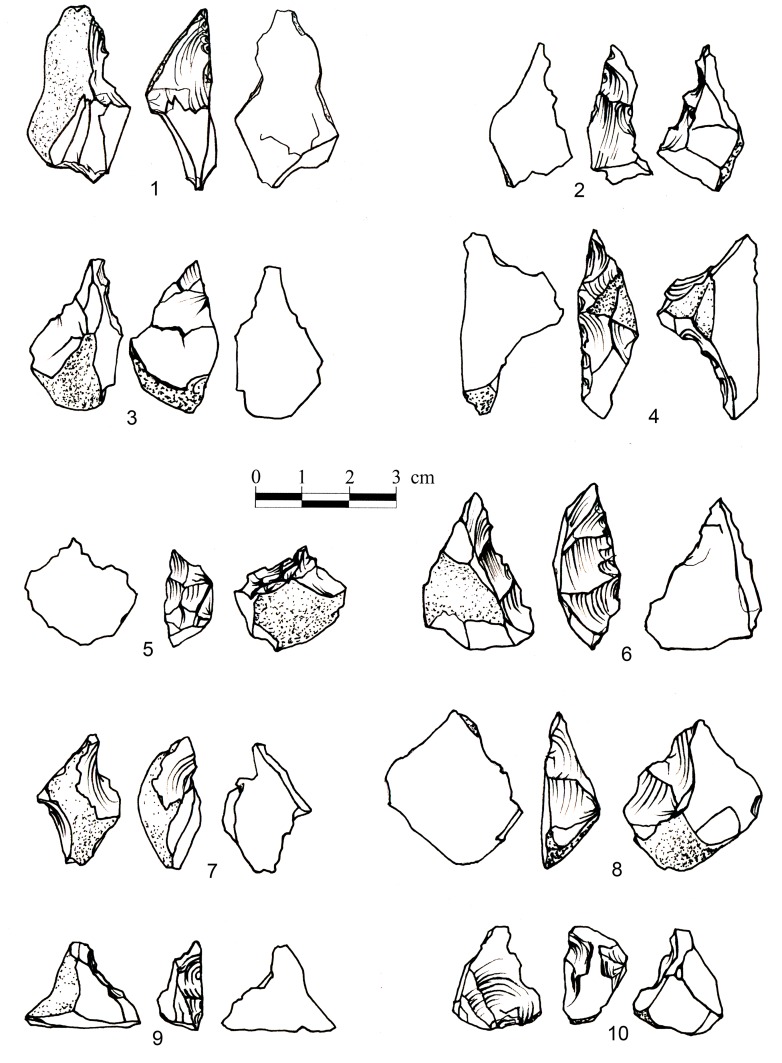
Experimental assemblages. **Secondary knapped flakes.** The “retouch” was accidentally produced by an anvil impact. Pieces 1,2,3,4,5,8,10 exhibit signs of the hammerstone impact on the dorsal faces. Morphology: 2,3,5,6,9,10– Pointed pieces; 1,7– Clactonian notches; 8– Flake with retouch-like scars.

**Figure 9:**
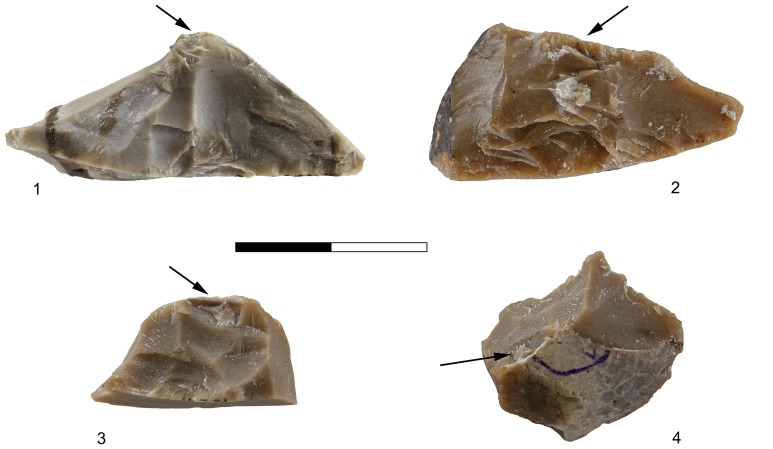
Experimental assemblages. **Secondary knapped flakes with signs of impact on the dorsal surface.** 1– Clactonian notch; 2, 3– Flakes with retouch-like scars; 4– Pointed piece. Arrows mark the signs of impact on the dorsal surface.

During the experiments a series of flakes was produced by a sequence of 3–4 blows. The detachment of the flakes often formed scars in the form of Clactonian notches or of retouch by large removals (Figures 7; 8; 9). Small spontaneous flakes (smaller than 1 cm) were usually removed with the larger flakes. Together with features of conchoidal fracture, some of the small scars showed step terminations without visible negatives of the bulb of percussion. A combination of large and small scars often created continuous denticulate or even rectilinear edges. The number and type of these spontaneous scars depended on the angle of the flake edge, the extent of the contact area between the flake edge and the anvil, and the strength of the hammerstone blow. Similar features have been recorded in other experiments in which flakes were knapped by bipolar technique [25], [43]. Moreover, since the flakes were removed from different edges of the “parent” flake, the convergence of these edges often created protrusions on the extremity of the knapped flake that resembled thick awls, beaks, or Tayacian points (Figures 6b; 8: 2, 3, 5, 6, 7, 9, 10; 9∶4). During the reduction sequence, a single “parent” flake could undergo transformation into first a Clactonian notch, then a pointed piece, and finally a broken flake. The edges of the knapped flakes became progressively abrupt during knapping (Figure 6c). In these later stages of the sequence, the features associated with wedging initiation, i.e., scars with shear surfaces and step-like terminations, became more dominant. Most of the broken flakes with signs of opposite impacts were produced during this advanced stage of the reduction sequence.

**Figure 10:**
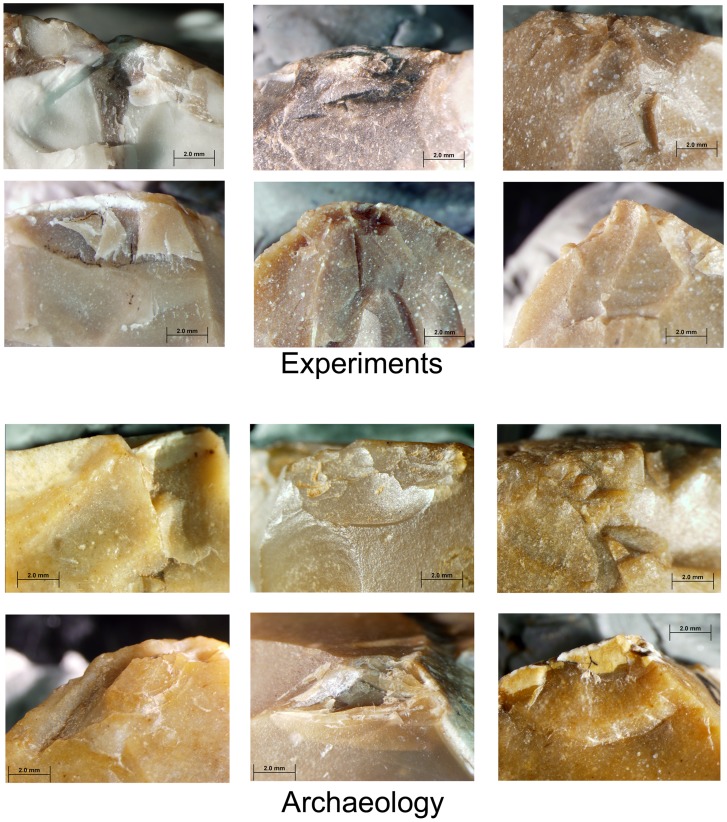
Signs of impact on the dorsal and lateral/broken surfaces intersection of secondary knapped flakes in archaeological and experimental assemblages.

#### The experimental assemblage

Altogether, Clactonian notches, flakes with retouch-like scars and broken flakes constitute 21.1% of the assemblage, while small flakes and fragments detached from their edges account for 78.9%. Each knapping sequence produced on average 2.18 artifacts of the first group, and 8.01 of the second. In total the 65 flakes that were knapped on an anvil yielded 667 artifacts (Table 2).

**Table 2:** The experimental assemblage breakdown.

Category	Subcategory	Number	% within a group	% from total
**Secondary knapped flakes**	Broken flakes with signs of dorsal impact	48	33.80%	7.20%
	Broken flakes without signs of dorsal impact	59	41.60%	8.80%
	Clactonian notches	14	9.90%	2.10%
	Flake with retouch-like scars	21	14.80%	3.10%
	**Sub-total**	**142**	**100.00%**	
**Detached pieces**	Small complete flakes	133	25.80%	19.90%
	Flake fragments	138	26.50%	20.70%
	Complete chips (<1 cm)	254	48.80%	38.10%
	**Sub-total**	**521**	**100.00%**	
**Total**		**667**		**100.00%**

Hammerstone impact marks occur on 54% of the flakes with retouch-like scars, 35.7% of the Clactonian notches and 45.8% of the broken flakes. The impact marks usually occur on the ridges at the contact between the dorsal and lateral or broken surfaces. They are localized and pronounced, indicating a small contact area. Crushing and small scars are the most frequent, followed by points and cones of percussion and then by various combinations of crushing marks, cones of percussion and wedge-shaped crack lines (Figures 9; 10; Table 3).

**Table 3:** The experimental assemblage.

	Broken flakes		Clactonian notches		Flakes with retouch-like scars		Pointed pieces		Total	
1. Point and/or cone of percussion	13	12.1%	1	7.1%	1	5.6%	1	33.3%	16	11.3%
2. Negative cone of percussion					1	5.6%			1	0.7%
3. Incipient cones					1	5.6%			1	0.7%
4. Dorsal face crushing	19	17.8%	2	14.3%	4	22.2%			25	17.6%
5. Wedge-shaped fracture lines	0	0.0%		0.0%		0.0%			0	0.0%
6. Combination of 1/2 and 4	7	6.5%	2	14.3%		0.0%			9	6.3%
7. Combination of 1/2+4+5	7	6.5%		0.0%	2	11.1%			9	6.3%
8. Combination of 4 and 5	2	1.9%		0.0%	1	5.6%			3	2.1%
9. No signs	59	55.1%	9	64.3%	8	44.4%	2	66.7%	78	54.9%
**Total**	**107**	**100.0%**	**14**	**100.0%**	**18**	**100.0%**	**3**	**100.0%**	**142**	**100.0%**

Signs of a hammerstone impact.

**Figure 11:**
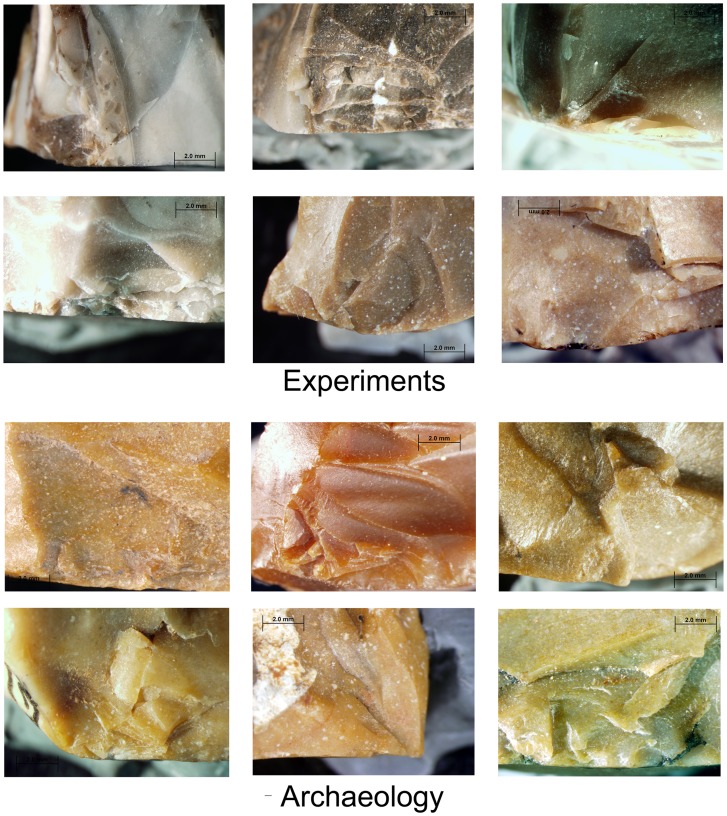
Signs of impact on the ventral and lateral/broken surfaces intersection of secondary knapped flakes in archaeological and experimental assemblages.

Anvil impact marks occur at various points along the circumference of the experimental items, indicating a large contact area between anvil and artifact. The anvil impact is evident on 59% of the experimental flakes that underwent further knapping. The anvil impact can be identified by the following:

- *Crushing*. These are most commonly found on broken flakes and are often associated with crack-lines, points of percussion and isolated scars (Table 4; Figures 9; 11).
- *Step fracture scars*. Flat, with no observable negatives of the bulb of percussion, these scars have step terminations. Step scars often occur in sequence and could be mistakenly identified as intentional abrupt retouch (Figures 9; 11). They may also occur in conjunction with conchoidal scars and crushing marks.
- *Conchoidal fracture scars*. When in sequence, choncoidal scars may resemble scaled retouch. Clactonian notches are large, wide conchoidal fracture scars with pronounced negative of the bulb of percussion.

**Table 4:** The experimental assemblage.

	Broken flakes		Clactonian notches	Flakes with retouch-like scars	Pointed pieces
1. Incipient cones	2	1.90%			
2. Broken surface scars and crushing	13	12.10%			
3. Isolated scars on lateral surface	1	0.90%			
4. Wedge-like crack lines	5	4.70%			
5. Point of percussion and crushing	6	5.60%			
6. Combinations of 1–4	22	20.60%			
7. No signs	58	54.20%			
8. Clactonian notches			14		1
9. Step-like fracture scars				9	
10. Conchoidal scars				3	
11. Combinations 8–10				6	2

Signs of an anvil impact.

Most experimental items exhibit more than one type of anvil impact marks (Table 4). Pointed pieces, for example, show a combination of step fracture scars and Clactonian notches.

**Figure 12:**
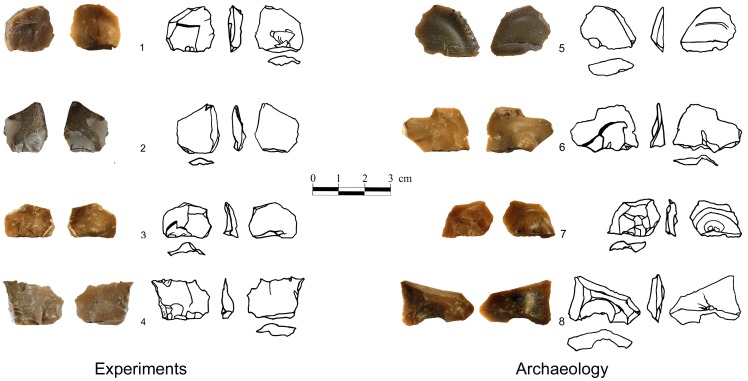
Small flakes from archaeological and experimental assemblages. Note the large bulbs of the specimens. The bulbs of experimental specimens were produced by an anvil impact.

A large number of small complete flakes were detached from the edges of the knapped flakes (Figure 12). The flakes show prominent bulbs of percussion, conchoidal fracture ripples and distinct ‘wing-shaped’ or lens-like butts (Figure 12). Small flakes are standardized in form and size, as well as in the shape of the butt and the bulb of percussion. They are thin, usually with sharp lateral and distal edges and one blunt edge formed by the butt. The length of the flakes is limited by the thickness of the selected blanks, while their butts are long and thick because the contact area between the anvil and the flake is large and not limited to one defined spot as is the case with hammer percussion (Figure 6a). The thickness and the acute angle between the ventral and lateral edges of the blanks seem to be another factor that facilitates the removal of flakes with a thick butt (Figure 6). The crack that forms the detached flake is propagated parallel to the edge of the ‘parent’ flake and terminates at its dorsal surface (Figure 6c). As a result, the detached flakes mostly exhibit feather or axial terminations. Hinge terminations are very rare. Impact marks of the hammerstone – points of percussion, wedge-shape crack lines and crushing – are visible on 26% of the flakes.

### Archaeological vs. Experimental Data

Clactonian notches, irregularly ‘retouched’ flakes, pointed pieces and broken flakes in the archaeological and experimental assemblages are similar in general morphology and in the shape of the scars along their edges (Tables 4; 5; Figures 3; 4; 8; 9; 11). The main difference between the archaeological and experimental assemblages is in higher frequencies of broken flakes in the latter (Tables 1; 2). There are two possible explanations for this difference. Firstly, the experimental assemblage contains broken flakes without signs of dorsal impact (Tables 2; 3). In archaeological sites similar breakage can result from knapping accidents, use, or depositional processes. Therefore, in archaeological assemblages only flakes with signs of dorsal impact were identified as secondary knapped flakes. Secondly, during the experiments the majority of the flakes was knapped until they broke. Shorter knapping sequence would have resulted in a considerably higher frequency of Clactonian notches and flakes with retouch-like scars.

The marks of the hammerstone impact on the experimentally-produced flakes are similar to the dorsal impact marks on the archaeological artifacts (Figures 4; 9; 10; Tables 3; 6). The same types of impact marks occur in both assemblages with some differences in the frequencies of the combinations of different marks. Slight variations in manufacturing techniques, e.g. changing the weight of the hammerstones or the force of the blow may be responsible for this discrepancy (see also [23]).

**Figure 13:**
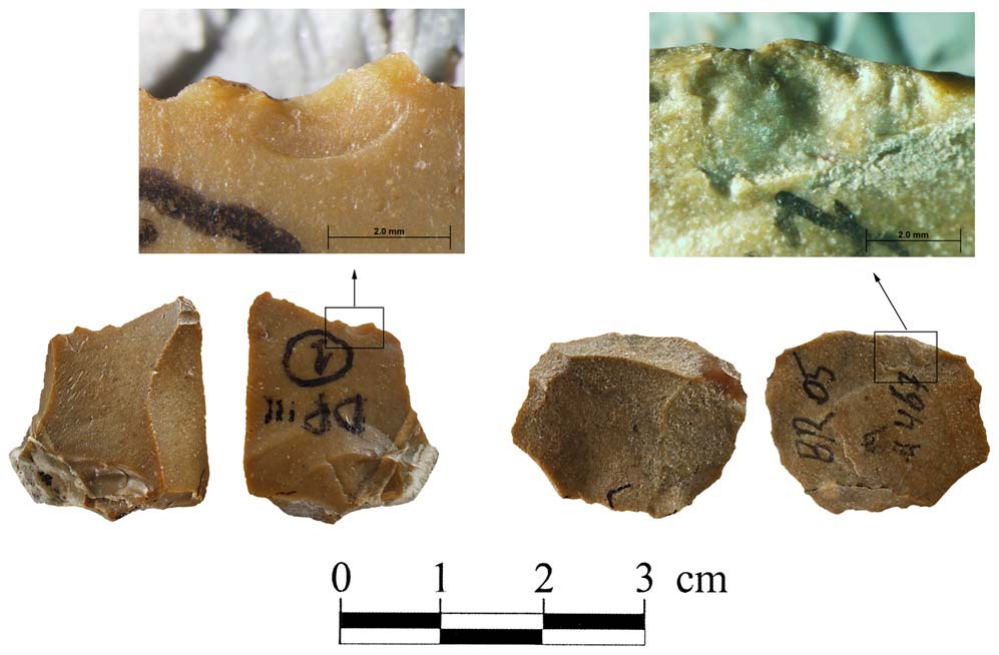
Small flakes from archaeological assemblages with signs of impact on the distal edges. The signs were probably caused by a hammerstone impact.

Small thin flakes that were produced during the experiments are similar in size and general morphology, as well as in the shape of the butt, to some of the small flakes that were found in the archaeological assemblages (Figures 12, 13; Table 7). These flakes were found in large frequencies in one of the excavated areas of the site (BRAT5 -19.5%), but were rare in another (BR1996–2%), possibly due to varying degrees of winnowing during the burial of the different areas of the site [19]. The majority of the small flakes from archaeological assemblages (87%) are complete and 79% of these exhibit pronounced bulbs of percussion. The butts are often large and have a wing-like or lens-like shape (Figure 12). Small flakes are thin and sharp with an angle of 25–55 degrees between the lateral and distal edges (mean –37.37, Std. D. –7.046). Eleven percent of these flakes in the archaeological assemblages exhibit marks of distal impact similar to the impact resulted from the hammerstone blows during the experiments (Figure 13).

**Table 5:** Archaeological assemblages.

	Broken flakes		Clactonian notches	Flakes withretouch-like scars	Pointed pieces
1. Incipient cones	2	1.1%			
2. Broken surface scars and crushing	39	22.0%			
3. Isolated scars on lateral surface	27	15.3%			
4. Wedge-like crack lines	7	4.0%			
5. Point of percussion and crushing	2	1.1%			
6. Combinations of 1–4	63	35.6%			
7. No signs	37	20.9%			
8. Clactonian notches			157		22
9. Step-like fracture scars				70	
10. Conchoidal scars				38	5
11. Combinations 8–10			61	198	106

Signs of an anvil impact.

**Table 6:** The archaeological assemblages.

	Broken flakes		Clactonian notches		Flakes with retouch-like scars		Pointed pieces		Total	
1. Point and/or cone of percussion	31	18.1%	5	2.3%	5	2.1%	5	3.8%	46	6.1%
2. Negative cone of percussion	27	15.8%		0.0%	3	1.3%		0.0%	30	3.9%
3. Incipient cones	2	1.2%	1	0.5%		0.0%		0.0%	3	0.4%
4. Dorsal face crushing	41	24.0%	15	6.9%	17	7.1%	14	10.5%	87	11.4%
5. Wedge-shaped fracture lines	6	3.5%		0.0%	2	0.8%		0.0%	8	1.1%
6. Combination of 1/2 and 4	41	24.0%	14	6.4%	12	5.0%	5	3.8%	72	9.5%
7. Combination of 1/2+4+5	11	6.4%	2	0.9%	9	3.8%		0.0%	22	2.9%
8. Combination of 4 and 5	12	7.0%	2	0.9%	4	1.7%	5	3.8%	23	3.0%
9. No signs			179	82.1%	186	78.2%	104	78.2%	469	61.7%
**Total**	**171**	**100%**	**218**	**100%**	**238**	**100%**	**133**	**100%**	**760**	**100%**

Signs of a hammerstone impact.

**Table 7:** Descriptive statistics of small flakes from experimental and archaeological assemblages.

		Length	Width	Thickness	Length of the butt	Thickness of the butt	Angle of the edge
Archaeology	N	151	151	154	144	145	151
	Mean	12.87	14.07	4.14	11.04	3.85	37.37
	Std. D.	2.759	3.784	1.254	4.954	1.771	7.05
Experiments	N	98	97	108	93	93	108
	Mean	12.17	14.7	4.17	11.28	3.59	36.33
	Std. D.	3.28	4.35	1.33	5.26	1.52	6.32

## Discussion

The experimental replication of the stone-tool production sequence at Bizat Ruhama supports the hypothesis that flakes were knapped on an anvil. The experiments demonstrate that the entire spectrum of edge modification morphologies identified at the site could have been produced by anvil impact during bipolar knapping of the flakes. During knapping, small chips and microflakes that resemble what Newcomer [24] identified as “spontaneous retouch” were removed simultaneously with larger flakes that often resemble Clactonian notches. The large and small scars often occur in succession, creating a denticulate or almost rectilinear edge that could be mistakenly identified as intentional retouch. Similar results have been obtained in other knapping experiments in which flakes were knapped by the bipolar technique [25], [43].

During the experiments, each knapping sequence produced a number of thin flakes 1–2 cm long. These flakes are the only standardized and systematic outcome of the secondary flake knapping and hence are the probable objective of the production. Judging from the experiments, the main reason for consistent detachment of uniform flakes was the use of thick steep-edged flakes as blanks for further knapping. The striking difference in the archaeological assemblages between the thickness values of flakes used as blanks for knapping and those of unmodified flakes indicates that the thick flakes were intentionally selected as blanks.

**Figure 14:**
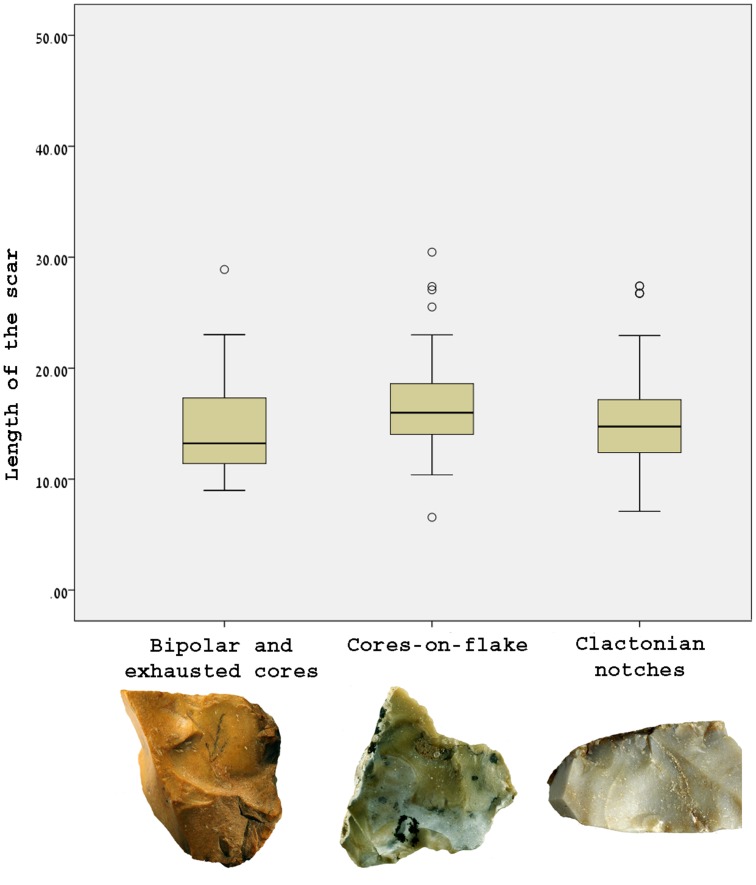
The size of the scars on cores-on-flake, bipolar and exhausted cores and Clactonian notches. The maximum length of the largest scar was measured.

**Figure 15:**
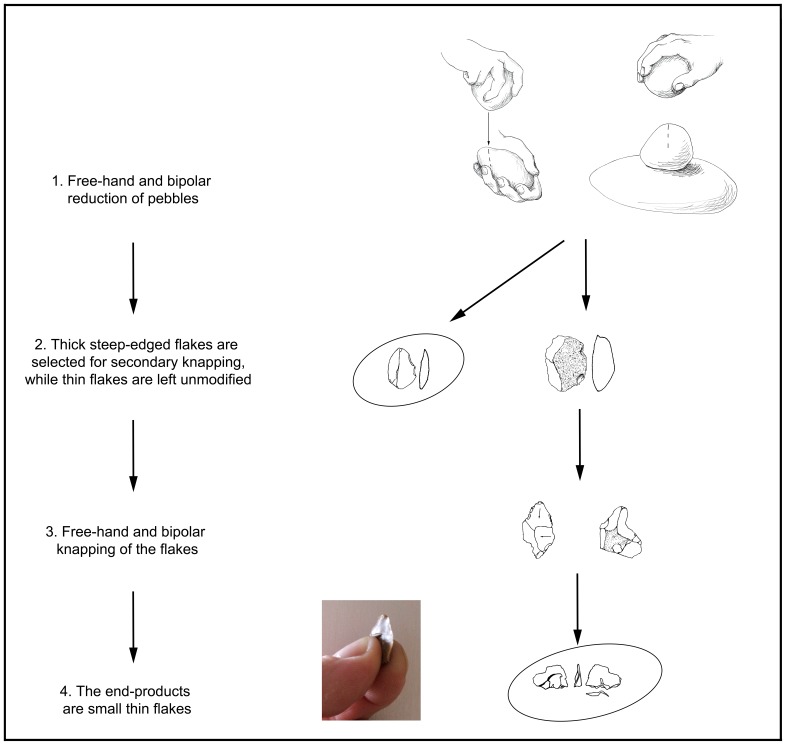
The lithic production scheme at Bizat Ruhama.

At Bizat Ruhama the production of small flakes was not restricted to a single operational scheme; the small flakes were also produced during cobble reduction and freehand secondary knapping of flakes. According to the size of the scars, all of these operational sequences yielded flakes of similar size (Figure 14). The lithic production system at the site was built of three successive stages (Figure 15):

1. Acquisition of raw material.
2. Production of flakes.
3. Further knapping of flakes.

This multi-stage operational sequence is linked by a relatively long succession of gestures, technical actions and decisions. The stage of flake production is characterized by simple core reduction methods identifiable with Oldowan technology. The unidirectional reduction method practiced at Bizat Ruhama is known from virtually every Oldowan assemblage from the earliest hominin sites at Gona, Ethiopia and Lokalalei 2C, Kenya to some of the later sites in the Olduvai Gorge, Koobi Fora and Sterkfontein [1], [6], [11], [30], [46], [47], [48], [49]. At 1.8–1.5 Ma, multifacial, unidirectional and multidirectional orthogonal knapping appeared across Oldowan assemblages in East Africa and led to the first occurrence of cores of polyhedral and subspheroid shape (e.g., [11], [30], [47], [50], [51]), similar to those resulting from orthogonal multifacial debitage at Bizat Ruhama. The bipolar technique, used so intensively at Bizat Ruhama, is known from many Oldowan sites, some of which are older than 2 Ma [41], [50], [52], [53], [54], [55]. The lithic industry of Bizat Ruhama shows no evidence for the production of large flakes which marks the emergence of the Acheulian in Africa [30], [56], [57]. Other advanced technological features, such as shaping, standardization of the tool’s shape, and bifacial and discoidal methods of core reduction, are lacking as well. In fact, alternating knapping either for preparation of the working edge or as part of chopper-like or discoidal flaking is not documented at the site and there is no evidence for the advanced platform preparation seen in some of the Oldowan and Early Acheulian assemblages at Olduvai Beds I and II, Peninj and Melka Kunture [30], [58], [59].

Two further components of the reduction system at Bizat Ruhama have not yet been reported from other Oldowan sites: systematic secondary knapping of flakes and the matching of a specific anvil technique to the flake knapping. The use of flakes as blanks for further reduction presumably reflects an adaptation of the Bizat Ruhama hominins to the small rounded pebbles available in the site’s vicinity [19], [20], [29]. The size of the pebbles and absence of good flaking angles hindered the initiation of knapping and the organization of core reduction. It is likely that the systematic secondary flake knapping and the application of the bipolar technique were part of a system developed by the Bizat Ruhama hominins in order to maximize exploitation of the lithic resources. The development of such a system reflects high adaptive flexibility and creativity and indicates that Oldowan hominins employed more complex operational schemes than has previously been suggested. In Oldowan sites, flakes were usually obtained directly from raw material nodules [2], [3], [4], [5], [6], [7], [11], [41], [46], [47], [49], [60], whereas flakes that were used as cores for removal of small flakes are extremely rare [1], [30], [47], [52], [60], [61]. Retouched artifacts are also rare in the Oldowan, and in recent studies of the assemblages from Olduvai Beds I and II by de la Torre and Mora [30] some of the items previously identified as retouched tools have been reclassified as natural pieces. Moreover, the bipolar technique that seem to have been an integral part of the technological repertoire of the Early/early Middle Pleistocene hominin populations in Africa and Eurasia [5], [7], [41], [42], [46], [47], [50], [52], [53], [54], [55], [62], [63], [64], [65], [66], was not used for the knapping of flakes. In several Oldowan sites in Africa where anvils were used and flakes of very small size occur, they were produced directly during pebble reduction (Sterkfontein; Fejej FJ-1a, Omo sites, Shungura Formation sites and Senga 5; [5], [46], [52], [53], [55], [63]. The small pebbles available at these sites were knapped by bipolar technique resulting in a large number of angular fragments and small flakes [5], [46], [50], [52], [54].

Only in the Middle Pleistocene did the secondary knapping of flakes become more frequent. In some late Lower and Middle Paleolithic sites, flakes were used as cores for removal of small flakes [67], [68], [69], [70], [71], [72], [73], [74]. In most of these cases the use of the bipolar technique is not documented, and breakage on an anvil has been suggested in only a few instances [23], [75], [76], [77], [78]. The secondary knapping of flakes in most of these cases reflects recycling and tool-kit maintenance (e.g. [23], [68], [73]). It is unclear whether reuse of flakes was part of the technological organization at Bizat Ruhama, or the use of flakes as blanks for further reduction was a stage in the primary conceptual framework of the lithic production, like the production of large flakes in the Acheulian. The intensity of the secondary knapping seems to speak in favor of the latter scenario.

Probably the closest parallel to Bizat Ruhama is the Middle Pleistocene site of Isernia La Pineta, Italy, where small flakes and fragments were produced during bipolar knapping of tabular flint nodules and flakes [25], [43], [79], [80], [81]. As at Bizat Ruhama, the Clactonian notches and denticulate edges that were often produced during the experimental knapping were interpreted as unintentional result of the production of small flakes. At Isernia La Pineta and the late Lower Paleolithic site at Qesem Cave, use-wear analysis indicates that thin flakes smaller than 2 cm were hand held and used for meat cutting [69], [79], [81].

The fact that the use of an anvil for knapping of flakes is not documented in other Early Pleistocene sites in Africa or Eurasia raises the question of whether this technique was invented by the Bizat Ruhama hominins. While systematic use of this reduction sequence seems to be unique to the site, evidence for use of similar techniques of flake knapping may possibly be found among Early Pleistocene retouched artifacts. Many of the retouched flakes in Early Pleistocene sites were formed by large Clactonian notches or irregular abrupt retouch that often created a pointed extremity (see [11]: appendices 6AA, 6DD, 6HH; [30]: figure 7.26; [49]: figure 4: 1; [51]: figures 5: 5–8; 6: 7–8; [58]: figure 10.3: 7–9; [82]: figures 8; 10: 3–4; 14:1–2; 24: 1– 2; 25: 1–2. At the site of Gombore 1 in Melka Kunture, Clactonian notches were frequently made on flakes and nodules of raw material [83]. In recent study of the assemblages from Olduvai Beds I and II [30] most of the retouched pieces and awls were reclassified as simple breaks, and Semaw et al. [57] has also suggested that most of the awls were unintentionally produced. At the Early Pleistocene site of Vallparadis, denticulate edges were interpreted as byproducts of knapping on an anvil [84]. Some of the cases noted above may have resulted from the sporadic use of the method identified at Bizat Ruhama.

### Conclusions

The operational sequence at Bizat Ruhama was more complex than that practiced at most Oldowan sites and included not only raw material acquisition and flake production but also the intentional selection of thick flakes and their further knapping on an anvil. The extent of secondary flake knapping at the site indicates that it played a fundamental role in the lithic production system. The probable goal of the flake knapping was to produce small, sharp flakes. The systematic secondary use of flakes for small flake production recorded at Bizat Ruhama was to become an integral part of lithic production systems hundreds of thousands of years later, from the end of the Lower Paleolithic onward e.g. [70], [71], [72], [74], [75], [76]. However, while at these sites secondary flake knapping was an element of recycling and maintaining the tool-kit, at Bizat Ruhama it seems to have had a more central function in the lithic reduction system, being one of the major sources of flake production. The use of this particular lithic production system highlights the early hominins’ capacity for invention and their adaptive flexibility, traits that probably played a major role in the out-of-Africa migration and the colonization of Eurasia.

Finally, this study advocates the need for caution when identifying intentional retouch and challenges the common belief that Clactonian notches and pointed artifacts were intentionally designed tools. The current study supports previous suggestions that Clactonian notches may be sources of small flakes rather than purposefully shaped tools [25], [70], [81]. This is especially true for the Early Pleistocene sites in which bipolar technique was frequently used.

## Supporting Information

Table S1 **Observations taken in course of the knapping experiments.**
(DOCX)

Table S2 **Full list of attributes applied to experimental and archaeological secondary knapped flakes.**
(DOCX)
