# Supplementary material for: Adaptive Flexibility of Oldowan Hominins: Secondary Use of Flakes at Bizat Ruhama, Israel
Source: PLoS One. 2013 Jun 20;8(6):e66851. doi: 10.1371/journal.pone.0066851 (PMC3689005; doi:10.1371/journal.pone.0066851)
Supplement: Table S1 — Observations taken in course of the knapping experiments. (DOCX) [file pone.0066851.s001.docx]

| Pre-knapping observations: | 1. Hammerstone size and weight 2. Length, thickness, width and angle of the lateral edges of the flakes |
| --- | --- |
| Observations made during the knapping | 1. Number of blows 2. Number of products produced by each blow 3. Recording the shape of the flake and the angle of lateral edges after each blow 4. Marking the direction of the hammerstone and anvil impacts on each product |
| Post-knapping observations | 1. Division of the assemblage according to the system used during the study of the archaeological material 2. Study of products applying the same list of technological attributes as during the study of the archaeological material 3. Description of the anvil impact signs 4. Description of the hammerstone impact signs 5. Description of the broken surface features |

**Table S**1. Observations taken in course of the knapping experiments.
