# Supplementary material for: Adaptive Flexibility of Oldowan Hominins: Secondary Use of Flakes at Bizat Ruhama, Israel
Source: PLoS One. 2013 Jun 20;8(6):e66851. doi: 10.1371/journal.pone.0066851 (PMC3689005; doi:10.1371/journal.pone.0066851)
Supplement: Table S2 — Full list of attributes applied to experimental and archaeological secondary knapped flakes. (DOCX) [file pone.0066851.s002.docx]

**Records on blanks**

| **Measurements** | **III. Butt** | **IV. Bulb** | **VIII. Edges** |  |  |
| --- | --- | --- | --- | --- | --- |
| Maximum length | Plain | Flat | Oval |  |  |
| Width | Cortical | Prominent | Parallel |  |  |
| Thickness | Dihedral | Indeterminate | Convergent |  |  |
| Butt length | Removed | With ridge | Divergent |  |  |
| Butt width | Broken | Pronounced cone | Irregular |  |  |
| Lateral edges angle | Indeterminate | Crushed | Indeterminate |  |  |
|  | Shattered |  |  |  |  |

***Measurements***

Maximum length is maximum distance between two points on the artifact's edges.

Width is measured at the widest point of the flake perpendicular to its technological axis.

Thickness is measured at the thickest point of the item.

Length of the butt is measured between lateral extremities of the butt.

Thickness of the butt is measured at the thickest part of the butt.

Angle of the edges is measured at the point where the edge shows lowest angle.

***Additional records taken for core-on-flakes***

| **Measurements** | **Scars location** | **Number of scars** |
| --- | --- | --- |
| Scar length  Scar width | Dorsal face  Ventral face  Both faces |  |

***Additional records taken for broken flakes***

| - Signs of impact on dorsal faces. - Signs of impact at the intersection between ventral and broken/lateral surface. - Shape of the scars at the intersection between the ventral and broken/lateral surfaces. - Broken surface features. |
| --- |

***Additional records taken for flakes with retouch-like scars***

| **A. Distribution*** | **B. Delineation** | **C. Angle of removals**** | **D. Scar morphology***** | **E. Edge modification type****** | **E. invasiveness** |
| --- | --- | --- | --- | --- | --- |
| Continuous | Rectilinear | Flat | Conchoidal fracture | Scaled | Long |
| Partial | Convex | Semi-abrupt | Clactonian notch | Step-like fracture | Short |
| Discontinuous | Concave | Abrupt | Step-like fracture | Marginal |  |
| Indeterminate | Notch | Cross-abrupt | Marginal | Irregular |  |
|  | Denticulate |  |  | Isolated removals |  |
|  | Irregular |  |  | Clactonian notch |  |
|  | Indeterminate |  |  | Clct. Notch and removals |  |
|  | One tooth |  |  |  |  |

* Continuous trimming – scars that cover at least 2/3 or of one of the flake edges

Partial trimming – scars that cover less than 2/3 of the edge

Discontinuous – scars that occur on different edges, in each case covering less than 2/3 of the flake edge

** flat - ≤ 45°; semi-abrupt 45°-70°; abrupt <90°; cross-abrupt ≥ 90

*** Conchoidal fracture – scars that show signs of conchoidal fracture, e.g. negative of the bulb of percussion, ripples.

Step-like fracture – scar that terminates as wide step and shows no features of conchoidal fracture, namely no impact point, no negative of the bulb of percussion and no ripple marks

Marginal scars – small and short scars on the flake edges (usually smaller than 3 mm in length and width)

**** Scaled – sequence of conchoidal fracture scars

Step-like fracture – sequence of step-like fracture scars

Irregular – combination of different types of scars

***Additional records taken for small flakes detached from secondary knapped flakes edges***

| **Distal end features** |
| --- |
| Scars on ventral face |
| Fracture lines |
| Crushing |
| Opposite bulbs |
| Combination of 1/2/3 |
| Hinge |
| Overshot |
